# IL-17A Synergizes with IFN-γ to Upregulate iNOS and NO Production and Inhibit Chlamydial Growth

**DOI:** 10.1371/journal.pone.0039214

**Published:** 2012-06-20

**Authors:** Yongci Zhang, Haiping Wang, Jianyun Ren, Xiaofei Tang, Ye Jing, Donghong Xing, Guosheng Zhao, Zhi Yao, Xi Yang, Hong Bai

**Affiliations:** 1 Department of Immunology, Tianjin Key Laboratory of Cellular and Molecular Immunology and Key Laboratory of Educational Ministry of China, School of Basic Medical Sciences, Tianjin Medical University, Tianjin, China; 2 Department of Clinical Laboratory, the Chinese People’s Liberation Army No.464 Hospital, Tianjin, China; 3 Laboratory for Infection and Immunity, Departments of Medical Microbiology and Immunology, Faculty of Medicine, University of Manitoba, Winnipeg, Manitoba, Canada; University of Bern, Switzerland

## Abstract

IFN-γ-mediated inducible nitric oxide synthase (iNOS) expression is critical for controlling chlamydial infection through microbicidal nitric oxide (NO) production. Interleukin-17A (IL-17A), as a new proinflammatory cytokine, has been shown to play a protective role in host defense against *Chlamydia muridarum* (Cm) infection. To define the related mechanism, we investigated, in the present study, the effect of IL-17A on IFN-γ induced iNOS expression and NO production during Cm infection in vitro and in vivo. Our data showed that IL-17A significantly enhanced IFN-γ-induced iNOS expression and NO production and inhibited Cm growth in Cm-infected murine lung epithelial (TC-1) cells. The synergistic effect of IL-17A and IFN-γ on Chlamydia clearance from TC-1 cells correlated with iNOS induction. Since one of the main antimicrobial mechanisms of activated macrophages is the release of NO, we also examined the inhibitory effect of IL-17A and IFN-γ on Cm growth in peritoneal macrophages. IL-17A (10 ng/ml) synergizes with IFN-γ (200 U/ml) in macrophages to inhibit Cm growth. This effect was largely reversed by aminoguanidine (AG), an iNOS inhibitor. Finally, neutralization of IL-17A in Cm infected mice resulted in reduced iNOS expression in the lung and higher Cm growth. Taken together, the results indicate that IL-17A and IFN-γ play a synergistic role in inhibiting chlamydial lung infection, at least partially through enhancing iNOS expression and NO production in epithelial cells and macrophages.

## Introduction

*Chlamydia trachomatis*, as an obligate intracellular bacterium, causes a wide variety of human and animal diseases including ocular, pulmonary, and genital infections. Strong Th1 responses marked by IFN-γ production are crucial for controlling chlamydial infection [1]. IFN-γ mediates antimicrobial action by activation of phagocytes for rapid uptake and degradation of pathogenic particles, and by inducing cellular enzymes, such as indoleamine 2, 3-dioxygenase (IDO), which cause tryptophan deprivation [2], [3] and inducible nitric oxide synthase (iNOS) which promote microbicidal nitric oxide (NO) production [1], [4]. It has been reported that iNOS rather than IDO is important for chlamydial control in mice [1], [5]. IFN-γ R^−/−^ or iNOS^−/−^ mice show higher pulmonary levels of chlamydial mRNA in comparison with wild-type (WT) mice after chlamydial infection [6], [7]. IFN-γ can also act directly on epithelial cell to inhibit the intracellular growth of *Chlamydiae* in vitro through inducing NO production [1]. However, uncontrolled NO release has also been implicated in inflammation. Therefore, proper modulation of iNOS-mediated NO release is important for therapeutic optimization of protective immunity and prevention of detrimental effects of inflammation during chlamydial infection [8], [9].

**Figure 1:**
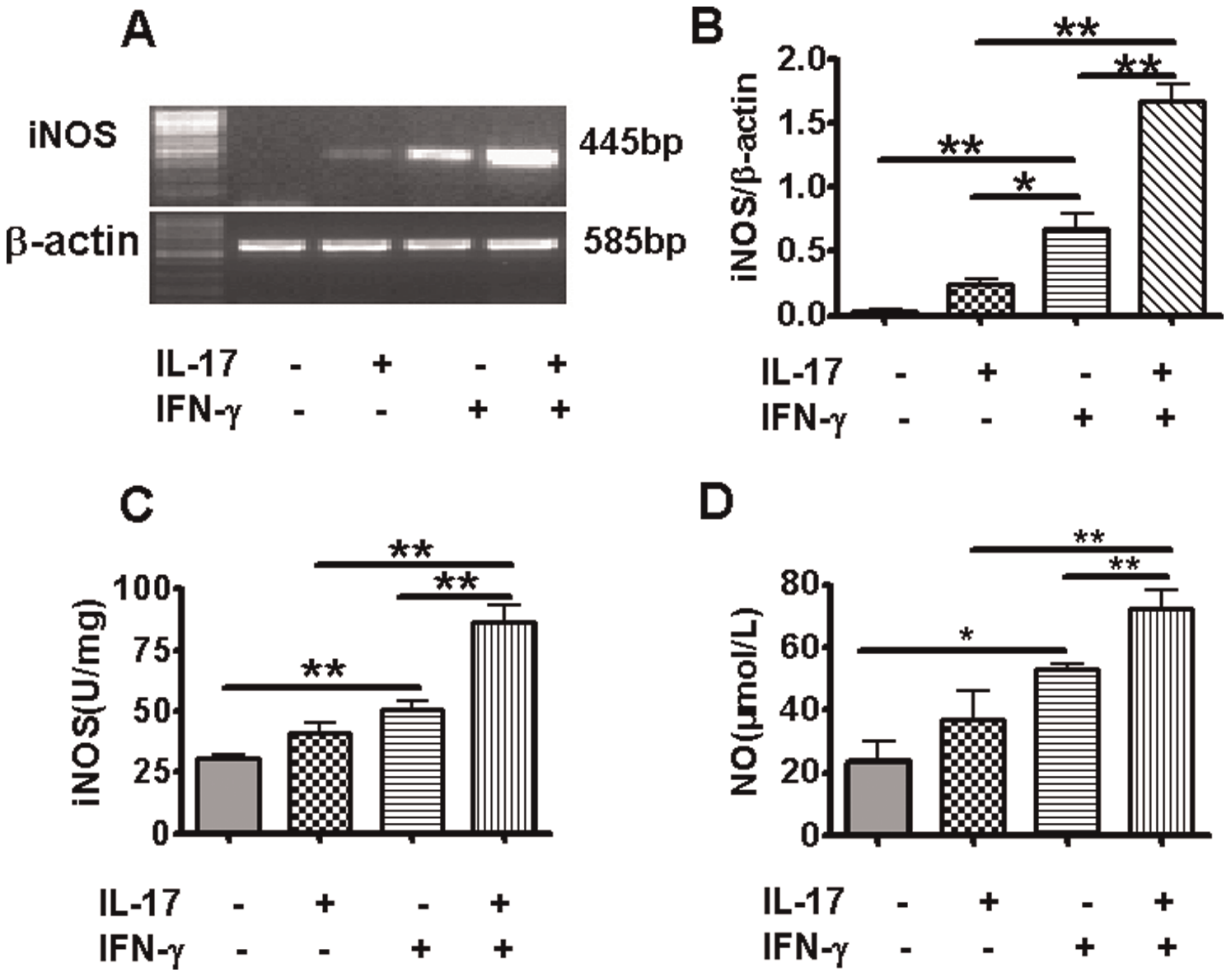
Effect of IL-17A and IFN-γ on iNOS gene expression, iNOS activity and NO production in TC-1 cells. TC-1 cells were cultured and treated with rmIL-17A (10 ng/ml) and/or rmIFN-γ (20 ng/ml) for 6 h. Cells were harvested, RNA was isolated and the mRNA level of iNOS was measured by RT-PCR. β-actin was used as an internal control. The representative gels were presented (A). The values of iNOS expression were normalized to β-actin and expressed as the mean ± SD of triplicate measurements (B). iNOS activity of cell lysates was detected by the chromatometry method (C). NO concentration in cell culture supernatants was detected using NO nitrate reductase method (D). *P<0.05, **P<0.01.

Interleukin-17A(IL-17A) is mainly produced by activated CD4^+^ T cells (Th17) and some other innate immune cell types such as γδT cells and NK1.1^−^ invariant NKT cells [10], [11], [12], [13]. Some data have proved that Th17/IL-17A is involved in host defense against intracellular bacterial infections, such as, mycobaterial infection through neutrophil induction and Th1 enhancement in mice [10]. Our previous study also showed that IL-17A/Th17 played an important role in defense against *Chlamydia* lung infection through modulating DC function and augmenting Th1 response [14]. Recently, IL-17A has gained increasing attention as a mediator of iNOS expression in a variety of cell types, such as human chondrocytes and murine osteoblasts, endothelial and beta cells which are involved in pathogenesis of some autoimmune diseases [15], [16]. Our previous study has shown that IL-17A-neutralized mice exhibited significantly higher chlamydial growth in the lung than sham-treated control mice [14]. Considering the importance of iNOS in controlling chlamydial infection, we hypothesized that IL-17A is involved in inhibition of intracellular Cm growth through enhancing iNOS expression and NO production.

In the present study, we first investigated the effect of IL-17A on iNOS expression in murine pulmonary epithelial cell line TC-1 and macrophage cell line RAW264.7 and found up-regulation of iNOS by IL-17A. Further experiments showed that IL-17A alone or in synergy with IFN-γ has a direct inhibitory effect on Cm growth in the pulmonary epithelial cell and peritoneal macrophages of mice. Finally, through adding of AG (a potent inhibitor of iNOS) into the system, we confirmed that the synergy of IL-17A and IFN-γ in inhibition of intracellular Cm growth is at least partly mediated by up-regulation of iNOS expression and NO production.

**Figure 2:**
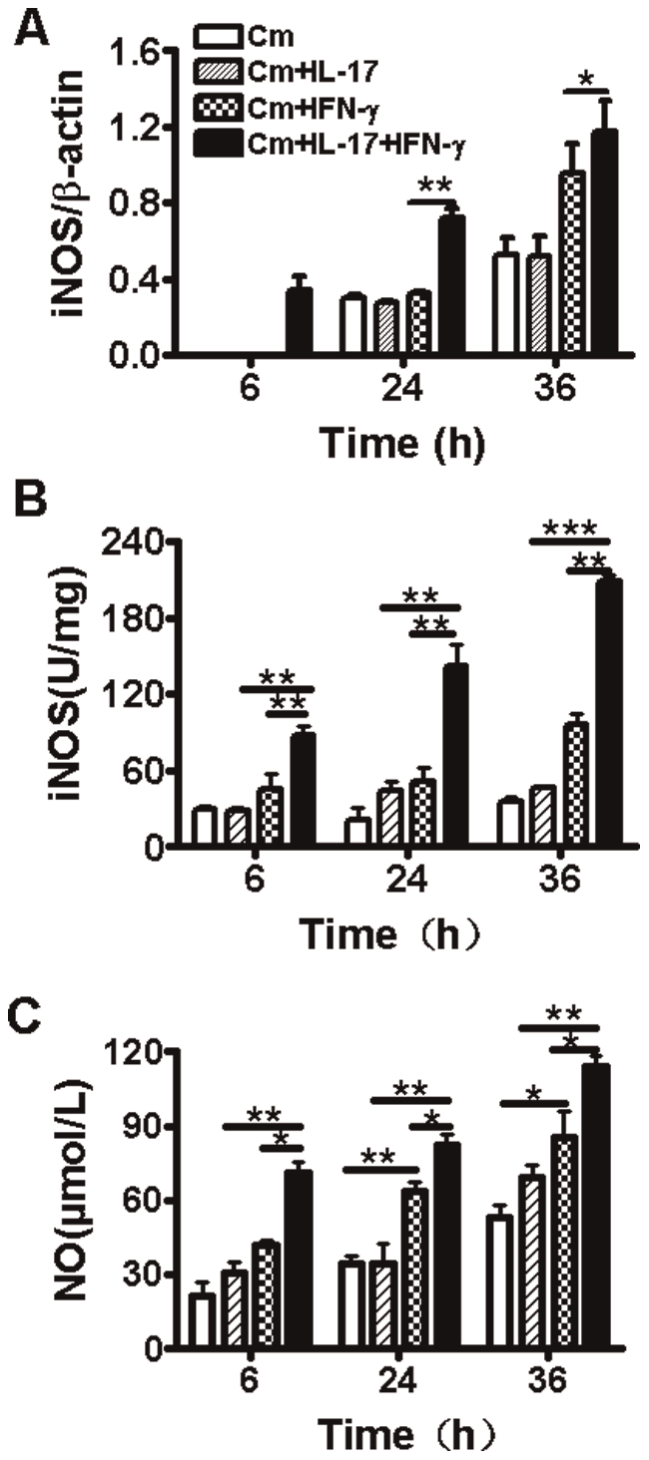
Effect of IL-17A and IFN-γ on iNOS gene expression, iNOS activity and NO production at various times following Cm infection. Cultured TC-1 cells were infected with Cm at MOI 2 and treated with rmIL-17A (10 ng/ml) and/or rmIFN-γ (20 ng/ml) at 2 h post-infection. The cells were harvested at 6, 24 and 36 h after infection and RNAs were isolated. The mRNA level of iNOS was measured by RT-PCR (A). The values of iNOS expression were normalized to β-actin. The results were presented as the mean ± SD of triplicate measurements. iNOS activity of cell lysate was detected by chromatometry method (B). NO concentration in cell culture supernatants was detected using NO nitrate reductase method (C). *P<0.05, **P<0.01, ***P<0.001.

## Materials and Methods

### Culture of TC-1 and RAW264.7

The murine lung epithelial cell line TC-1 and macrophage cell line RAW264.7 were obtained from American Type Culture Collection (ATCC). TC-1 and RAW264.7 cells were grown in 24-well plates at density of 3–5 million cells per well in RPMI 1640 medium with 10% FBS. Before treatment with cytokines, cells were incubated in RPMI 1640 medium with low (0.5%) FBS without penecilin/streptomycin overnight to starve cells for more efficient taken up of cytokines by the cells. Cells were treated with 10 mg/ml mouse recombinant IL-17A (rmIL-17A) or 20 ng/ml mouse recombinant IFN-γ (rmIFN-γ) (both were obtained from PeproTech Inc.) in various combinations for 6 h. Endotoxin levels in commercial rmIL-17A, rmIFN-γ and rmTNF-α were less than 0.1 ng in 1 µg of the cytokine. Cells were harvested for iNOS mRNA expression assay and iNOS activity assay, while the cell culture supernatants were harvested for NO production detection.

**Figure 3:**
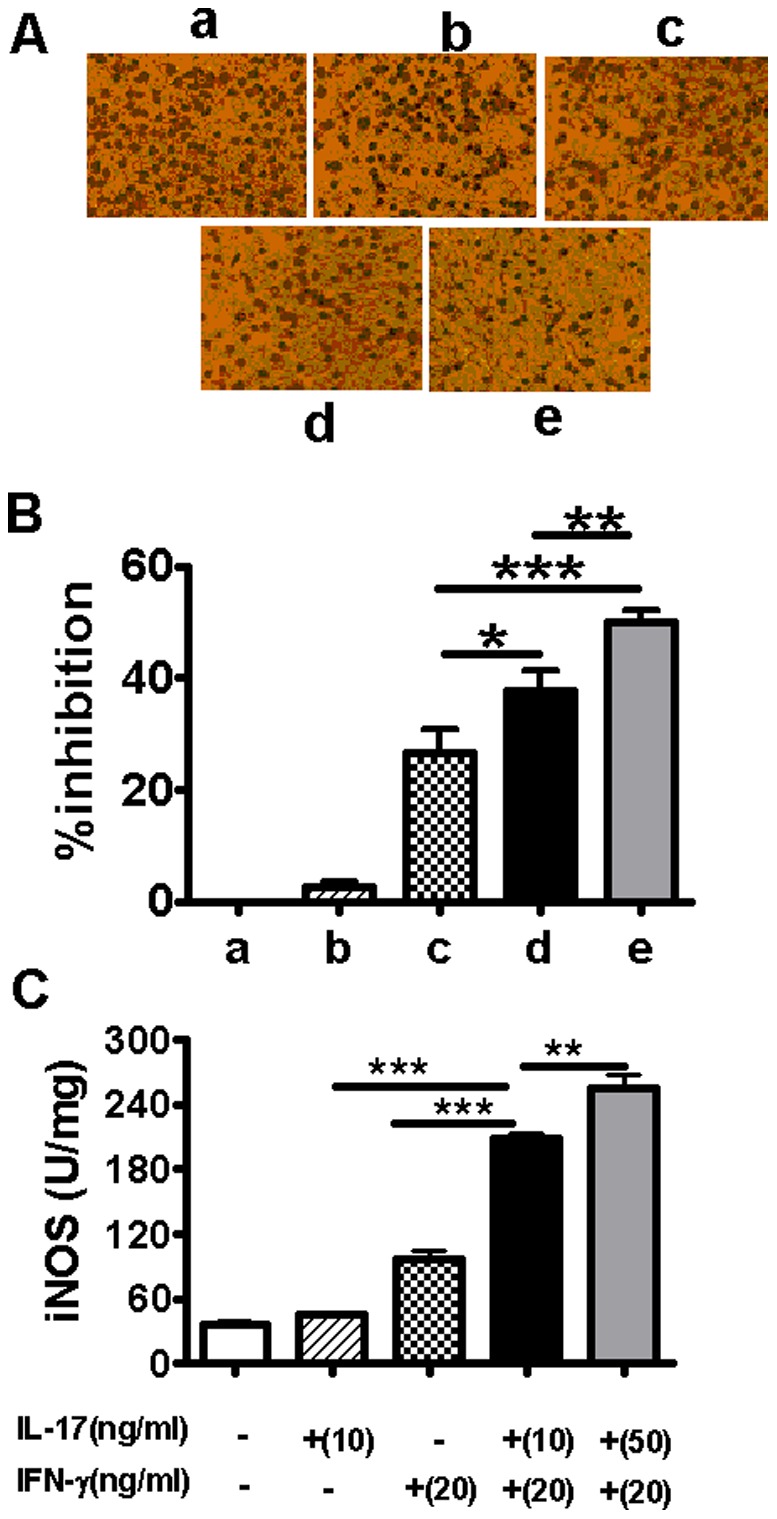
Inhibition of IL-17A and IFN-γ on intracellular Cm growth in TC-1 cell. TC-1 cell monolayers were infected with Cm and treated with rmIL-17A (10 or 50 ng/ml) and/or rmIFN-γ (20 ng/ml) at 2 h post-infection. Cm inclusions in the infected TC-1 cells at 36 h after infection were stained with anti-chlamydial LPS mAb. Cm was quantified as inclusion forming units (IFUs)/ml. The representative pictures of Cm inclusions in different groups were shown (A). The percentage of inhibition was calculated as described in the Materials and Methods (B). iNOS activity of cell lysate was detected by chromatometry method (C). The results were expressed as the mean ± SD of triplicate measurements. *P<0.05, **P<0.01, ***P<0.001.

### Infection of TC-1 Cells

*Chlamydia muridarum* (Cm), a natural mouse chlamydial strain, was propagated in Hep-2 cells (ATCC) as described previously [17]. TC-1 cells were seeded into 24- and 12- well plates at a density of 2–3×10^5^ cells/ml, and cultured for 24 h. Cell monolayers were washed twice with phosphate buffered saline (PBS) and infected with *Cm* diluted in RPMI 1640 medium. Plates were placed at 37°C in 5% CO_2_ incubator for 2 h and the extracellular bacteria were removed by washing with PBS. Subsequently, cells were cultured in fresh RPMI-1640 containing 0.5% FBS, in the presence or absence of rmIL-17A and/or rmIFN-γ at the indicated concentrations. The cells from 24-well plates were harvested for iNOS mRNA expression assay and iNOS activity assay at 6 h, 24 h and 36 h after infection, and the cell culture supernatants were harvested for NO production detection. Measurement of chlamydial growth in the wells of different combinations of cytokines was performed by staining of chlamydial inclusion in the 12-well plates at 36 h after infection using anti-chlamydial LPS mAb and was shown as inclusion forming units (IFUs)/ml as described previously [14]. The effect of rmIL-17A and rmIFN-γ on the inhibition of *Cm* growth was measured by inhibition rate as calculated below [1]:

**Figure 4:**
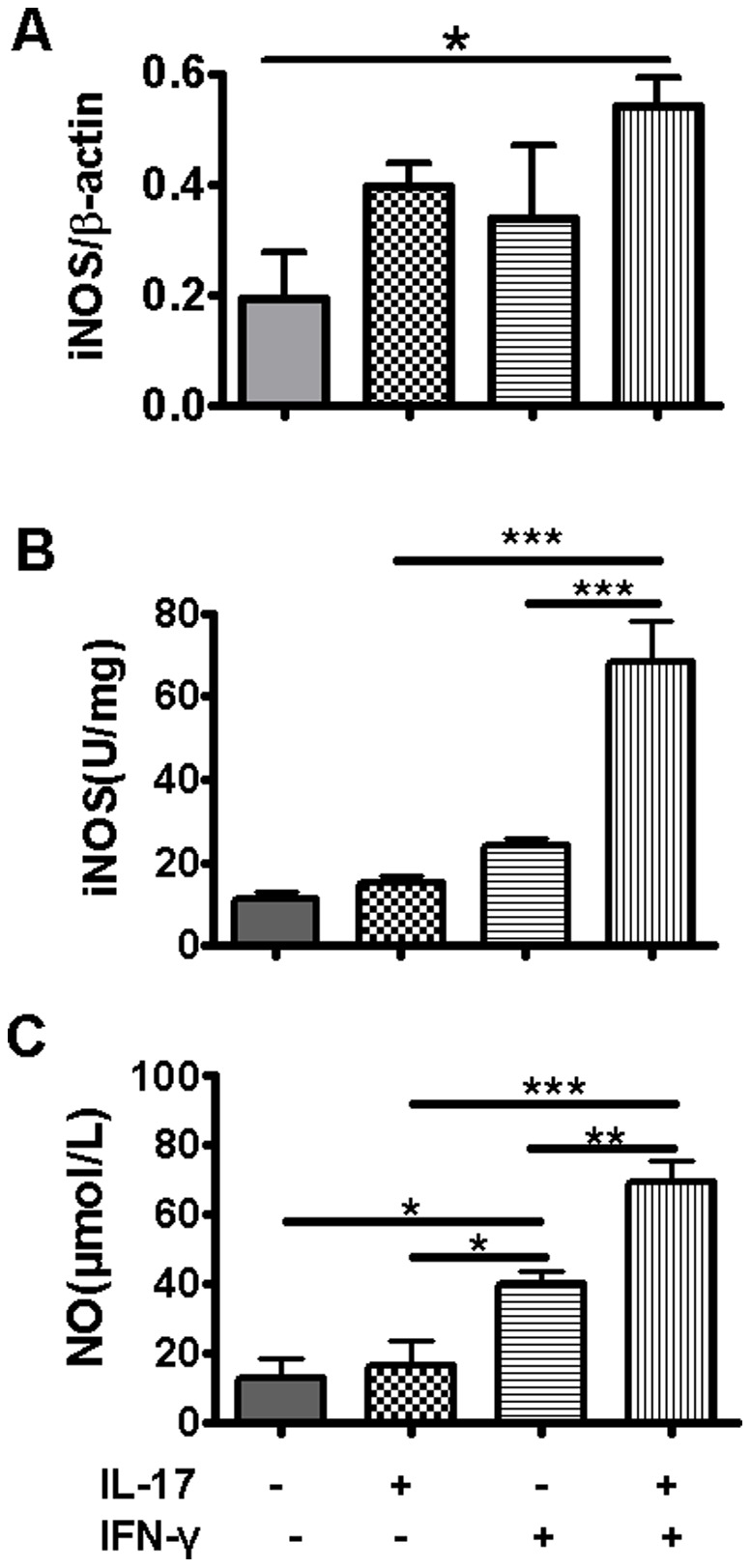
Effect of IL-17A and IFN-γ on iNOS gene expression, iNOS activity and NO production in RAW 264.7 cells. RAW 264.7 cells were cultured and treated with rmIL-17A (10 ng/ml) and/or rmIFN-γ (20 ng/ml) for 6 h. Cells were harvested, RNA was isolated and the mRNA level of iNOS was measured by RT-PCR. β-actin was used as an internal control. The representative gels were presented. The values of iNOS expression were normalized to β-actin and expressed as the mean ± SD of triplicate measurements (A). iNOS activity of cell lysates was detected by chromatometry method (B). NO concentration in cell culture supernatant was detected using NO nitrate reductase method (C). *P<0.05, **P<0.01, ***P<0.001.

Inhibition rate (%)  =  (mean IFUs/ml of control wells – mean IFUs/ml of experimental wells) ×100%/mean IFUs/ml of control wells.

### Infection of Macrophages with Cm in vitro

Peritoneal macrophages were collected from BALB/c mice following thioglycollate (4%, 1.5 ml/mouse) injection as described [18]. Peritoneal cells were harvested by lavaging the peritoneal cavity with 8 ml Hanks balanced salt solution (HBSS) 4 days after thioglycollate injection, followed by centrifugation (210 g, 10 min) of the lavage fluids. Following lysing red blood cells with 0.85% NH_4_Cl, the peritoneal cells (mostly macrophages) were resuspended at 2×10^6^ cells/ml with 10% FBS RPMI-1640 medium and were set into 96-well culture plates at 100 µl/well. Two hours after incubation at 37°C in a CO_2_ incubator, unattached cells were washed away with HBSS. Fresh complete culture medium (200 µl/well) was added into wells and the plates were further incubated overnight at 37°C to form cell monolayers. The macrophage monolayers were then inoculated with Cm for 2 h followed by washing with HBSS. After that, fresh complete culture medium with different concentrations of rmIFN-γ (10 or 200 ng/ml) and/or rmIL-17A (10 ng/ml or 100 ng/ml) were added to the wells at 200 µl/well. In the specified experiments, aminoguanidin (AG, Sigma) was added to the wells containing rmIFN-γ and/or rmIL-17A at a final concentration of 100 µM. Following 48 h culture, chlamydial inclusions in the macrophage monolayers were stained and the percentage inhibition was computed using the same formula shown above.

**Figure 5:**
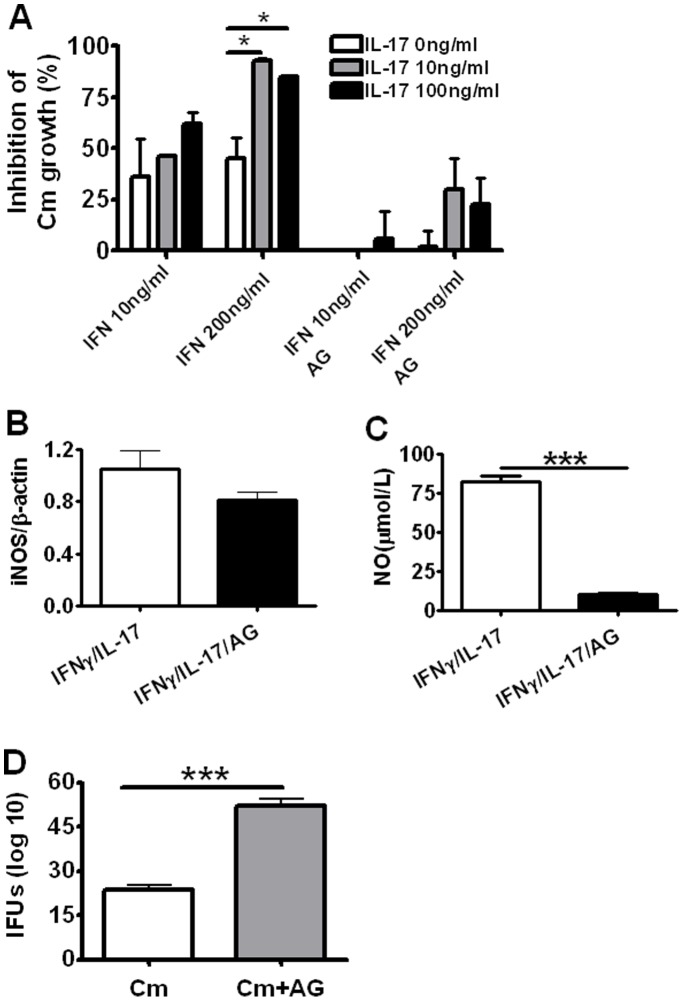
Inhibition of IL-17A and IFN-γ on intracellular Cm growth in cultured ex vivo murine macrophages. Peritoneal macrophages were collected from BALB/c mice following 4% thioglycollate injection. The macrophage monolayers were then inoculated with Cm for 2 hr followed by addition of different concentrations of rmIFN-γ (10 or 200 ng/ml) and/or rmIL-17A (10 ng/ml or 100 ng/ml). Aminoguanidin (AG) was added to the wells containing IFN-γ and/or IL-17A at a final concentration of 100 µM. Chlamydial inclusions in the macrophage monolayers were stained and counted and the percentage of inhibition was calculated as described in the Materials and Methods (A). The cells were harvested and RNA was isolated. The mRNA level of iNOS was measured by RT-PCR. The values of iNOS expression in IFN-γ and IL-17A treated cells with or without AG were normalized to β-actin (B). NO concentration in cell culture supernatant was detected using NO nitrate reductase method (C). The Cm growth (IFU) in Cm-infected macrophage in absence or presence of AG was assayed (D). The results were presented as the mean ± SD of triplicate measurements. *P<0.05. **P<0.01, ***P<0.001.

### Infection of Mice and Neutralization of Airway IL-17A

Female BALB/c mice, 6–8 week old, were kept in a specific pathogen-free facility at the central animal care facility in Tianjin Medical University with filtered air flow and autoclaved cage, food and water. Mice were anesthetized and inoculated intranasally with 1×10^3^ inclusion forming units (IFUs) of Cm in 40 µl final volume of PBS. For neutralization of airway IL-17A, mice were intranasally administered with 10 µg of anti-mouse IL-17A mAb (R&D) in 40 µl of PBS at 2 h postinfection (p.i.), followed by subsequent administration of the same dose of Ab at 48-h intervals. Infected control mice were administered intranasally with isotype control (IgG2a) antibody in the same schedule as anti-IL-17A delivery. Mice were killed at day 7 after infection and the lungs were isolated for gene expression assay and chlamydial growth as described [17]. All mice in this study were used in accordance with the guidelines issued by the Chinese Council on Animal Care and animal experimental protocol approved by the ethical committee of Tianjin Medical University (Approval Number: TY10-101).

**Figure 6:**
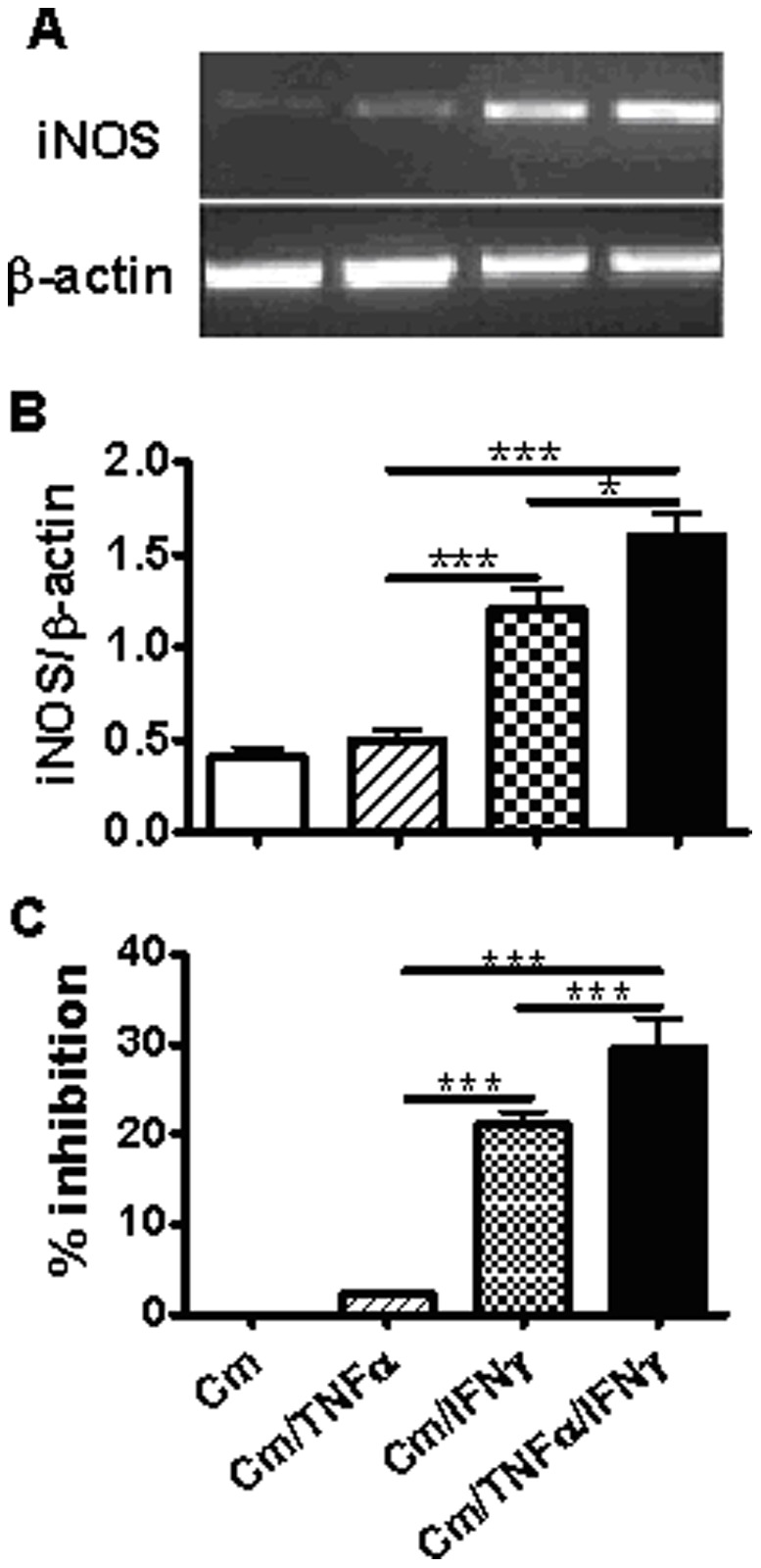
Effect of TNF-α and IFN-γ on iNOS gene expression and Inhibition of intracellular Cm growth in TC-1 cell. Cultured TC-1 cells were infected with Cm at MOI 2 and treated with rmTNF-α (10 ng/ml) and/or rmIFN-γ (10 ng/ml) at 2 h postinfection. The cells were harvested at 36 h after infection and RNAs were isolated. The mRNA level of iNOS was measured by RT-PCR (A). The values of iNOS expression were normalized to β-actin. The results were presented as the mean ± SD of triplicate measurements (B). Cm was isolated from infected TC-1 cells at 36 h after infection and Cm was quantified as IFUs/ml. The percentage of inhibition was calculated as described in the Materials and Methods(C). *P<0.05, ***P<0.001.

### RT-PCR

Transcriptase-polymerase chain reaction (RT-PCR) was used to assay iNOS mRNA expression. Total RNA was prepared using a Rapid RNA Extraction Kit (Bioteke Corporation, China) according to the manufacturer^’^s protocol. Total RNA (1 µg) was reverse-transcribed into cDNA which was then amplified using specific primers for iNOS and β-actin. The PCR primers used in this study were as follows: iNOS, 5′-CCCTTCCGAAGTTTCTGGCAGCAGCAGC-3′(sense) and 5′-GGCTGTCAGAGCCTCGTGGCTTTGG-3′ (anti-sense) to give a 445-bp product; β-actin, 5′-ATGGATGACGATATCGCT-3′ (sense) and 5′-ATGAGGTAGTCTGTCAGGT-3′(anti-sense) to give a 585-bp product. PCR conditions were as follows: iNOS and β-actin: 94°C 10 min, followed by 30 cycles of 94°C for 50 s, 55°C for 45 s and 72°C for 45 s. PCR products were electrophoresed on 1.5% agarose gel containing ethidium bromide (0.0003%). The bands were visualized and photographed using ultraviolet transillumination and were analysed for density on Quantity One software and normalized against β-actin signal from the same sample.

**Figure 7:**
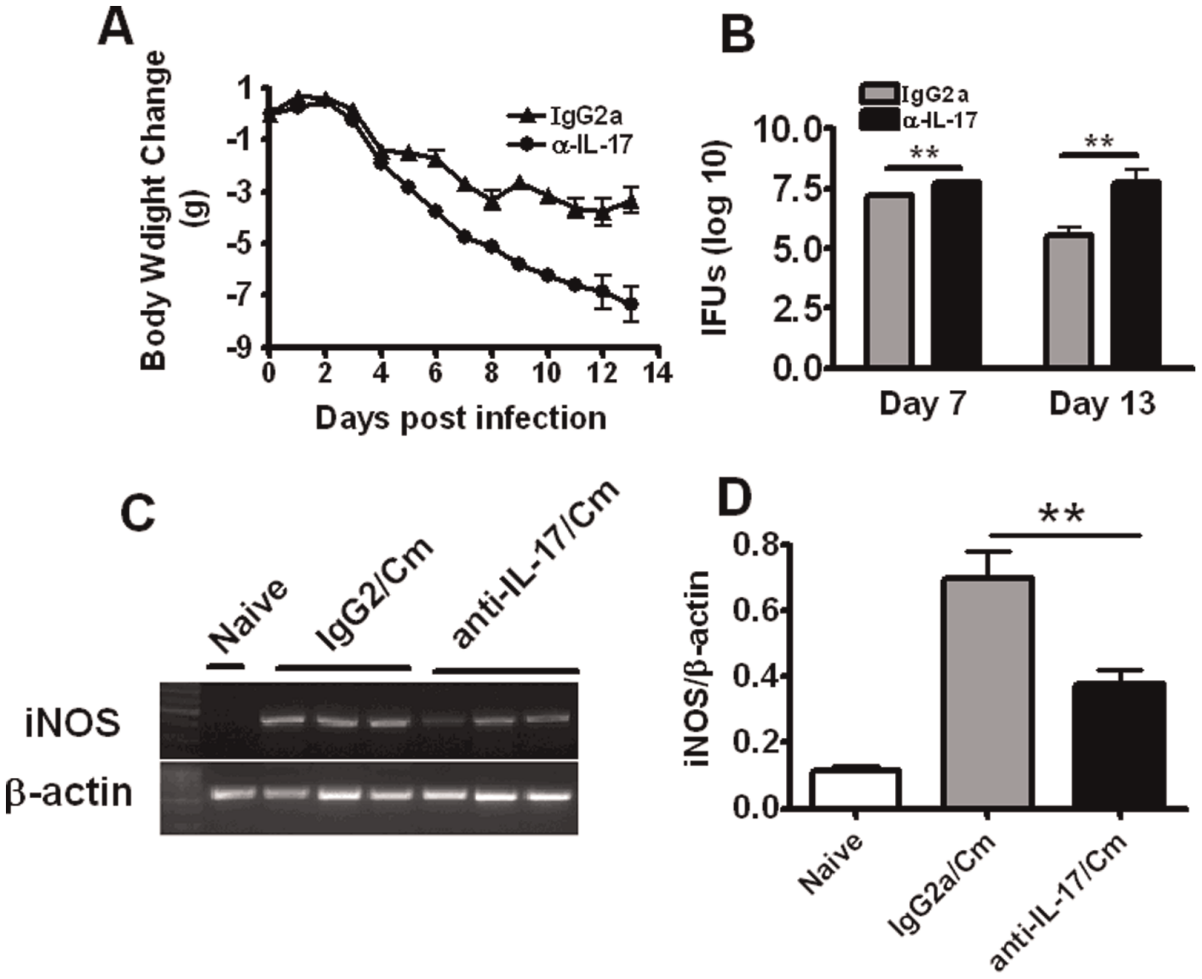
The comparison of iNOS expression between IL-17A-neutralized mice and IgG2a-treated control mice. Mice were intranasally infected with 1×10^3^ IFUs of Cm. For neutralization of airway IL-17A, mice were intranasally administered with 10 µg of anti-mouse IL-17A mAb in 40 µl of PBS at 2 h post-infection (p.i.), followed by subsequent administration of the same dose of Ab at 48-h intervals. Infected control mice were administered intranasally with anti-murine IL-17A Ab isotype control (IgG2a) in the same schedule as anti-IL-17A delivery. Mice were monitored for body weight changes (A) and killed at day 7 after infection. The lungs were collected and analyzed for in vivo chlamydial growth (B) and gene expression (C and D) as described in *Materials and Methods*. D, densitometric analysis of iNOS mRNA RT-PCR products from lung in mice at day 7 postinfection. Values were normalized to β-actin and expressed as the mean ± SD with n = 5 for each point. **, p<0.01.

### Detection of iNOS Activity in Cell Lysates

iNOS activity in cell lysates was detected using an Isoforms of Nitric Oxide Synthase Assay Kit (Nanjing Jiancheng Bioengineering Institute, Nanjing, China) according to the manufacturer’s protocol. Briefly, cell lysates were prepared by sonicating the cells in 0.5 ml of cold PBS and the protein concentrations were determined. Then, the absorbance of differential samples in 530 nm was detected by a chromatometry method. The iNOS activity was calculated according to the formula provided by the manufacturer of the Kit.

### Determination of NO Concentration in Cell Culture Supernatants

NO concentration in cell culture supernatants was detected using a NO nitrate reductase assay kit (Nanjing Jiancheng Bioengineering Institute, Nanjing, China) according to the manufacturer’s protocol. NO concentration was calculated according to the following formula: NO (µmol/L)  =  standard concentration(100 µmol/L)×Dilution factory of sample×(OD of NO measuring tube–OD of blank tube )/(OD of Standard – OD of Blank).

### Statistical Analysis

Statistical analysis was performed using SPSS 12.0 version software. For comparisons of more than groups, data were subjected to one-way ANOVA followed by the Student-Newman-Keuls multiple comparison test.

## Results

### IL-17A Synergizes with IFN-γ for Enhancing iNOS Expression and NO Production by TC-1 Cells in the Absence of Infection

IFN-γ-mediated antimicrobial action is mainly dependent on inducing cellular enzymes, such as inducible nitric oxide synthase (iNOS) to produce microbicidal NO [1], [4]. We first tested the effect of IL-17A and IFN-γ on iNOS and NO production in uninfected TC-1 cell, a murine lung epithelial cell line. The results showed that IL-17A alone had no effect on iNOS and NO production, but it significantly augmented IFN-γ-induced enhancement of iNOS mRNA expression (Fig. 1A and B), iNOS activity (Fig. 1C) and NO production (Fig. 1D).

### IL-17A Synergizes with IFN-γ for Enhancing iNOS and NO Production in Cm-Infected TC-1 Cells

We further evaluated the effects of IL-17A on iNOS mRNA expression, iNOS activity, and NO production in *Cm*-infected TC-1 cells. As shown in Fig. 2A, at 6 h after Cm infection, only the wells with combined IL-17A and IFN-γ stimulation showed measurable iNOS mRNA expression, but not those with single cytokine. At 24 h and 36 h after infection, the mRNA expression of iNOS were measured in all groups but the expression in the TC-1 cells treated by the combination of IL-17A and IFN-γ was significantly higher than others. No significant change of iNOS mRNA expression and iNOS activity was found in the wells with IFN-γ alone (Fig. 2A and 2B), but significant increase was found for NO production at 24 h and 36 h after infection (Fig. 2C). However, the combination of IFN-γ and IL-17A induced significantly higher levels of iNOS activity and NO production than IFN-γ alone in *Cm*-infected TC-1 cells at each time point after infection (Fig. 2B and C). The results suggest that IL-17A can synergize with IFN-γ to enhance IFN-γ-mediated iNOS induction and NO production during Cm infection.

### IL-17A Enhances the Inhibitory Activity of IFN-γ on Cm Growth in Pulmonary Epithelial Cells

After showing the synergy of IL-17A and IFN-γ in inducing iNOS activity and NO production in uninfected and Cm-infected TC-1 cells, we further examined whether this synergy led to enhanced inhibition of chlamydial growth. As shown in Fig. 3A and 3B, IL-17A (10 ng/ml) alone had no effect on inhibition of Cm growth in TC-1 cells at 36 h after infection, but it significantly enhanced the inhibitory activity of IFN-γ (20 ng/ml) in a dose-dependent manner. The synergistic inhibitory effect of IL-17A and IFN-γ on chlamydial growth in TC-1 cells correlated with iNOS induction (Fig. 3C). The combination of IL-17A with IFN-γ induced higher level of iNOS activity than IFN-γ alone in a dose-dependent manner at 36 h after infection. The results indicate that IL-17A can synergize with IFN-γ to inhibit Cm growth, which correlates with iNOS activity.

### IL-17A Synergizes with IFN-γ for Inducing iNOS and NO Production in RAW 264.7 Cells

It is known that one of the main antimicrobial mechanisms of activated macrophages is the release of NO [18],[19]. Using murine macrophage cell line RAW 264.7, we found that 20 ng/ml IFN-γ alone can induce significant NO production by RAW 264.7 cells, although no significant change in iNOS mRNA expression was detected at the measured time point (Fig. 4). However, IL-17A showed a significantly synergistic effect with IFN-γ on iNOS activity and NO production in murine macrophage cell line RAW 264.7 (Fig. 4 B and C).

### IL-17A with IFN-γ in an Appropriate Proportion Inhibited Cm Growth in Cultured ex vivo Macrophages from Mice

In order to test effect on primary cells, we further collected peritoneal macrophages from naïve BALB/c mice and tested the effect of IL-17A alone or in combination with IFN-γ on Cm growth. Isolated peritoneal macrophages were infected *in vitro* with Cm. Different concentrations (10 ng/ml or 200 ng/ml) of IFN-γ and/or IL-17A (10 ng/ml or 100 ng/ml) were added to the culture after inoculation with Cm. The control wells had Cm infection alone without other treatment. We found that IL-17A (10 ng/ml or 100 ng/ml) could synergize with high (200 ng/ml), but not low concentration (10 ng/ml), of IFN-γ for inhibiting intracellular Cm growth in macrophages (Fig. 5–A), In the low IFN-γ (10 ng/ml) concentration, the inhibition rates by both concentrations of IL-17A (10 ng/ml or 100 ng/ml) with IFN-γ were not significantly higher than IFN-γ alone (Fig. 5A). These results indicated that IL-17A could significantly inhibited Cm growth in macrophages in higher concentration of IFN-γ (200 ng/ml).

To directly test whether the synergistic inhibitory effect on Cm growth by IL-17A and IFN-γ is dependent on iNOS expression, we used AG, an analogue of L-arginine that is a potent inhibitor of iNOS activity, to block the function of iNOS to promote NO production and the effect on Cm growth. As shown in Fig. 5A, following cytokines stimulation, the inhibition of Cm growth by IL-17A and IFN-γ in the culture wells with AG was significantly lower than those wells without AG in different combinations and concentrations of these cytokines. Consistently, although the addition of AG had no significant effect on iNOS expression (Fig. 5B), it significantly lowered NO production (Fig. 5C) and Cm growth (Fig. 5D). The data indicate that the inhibitory effect on Cm growth by IL-17A in synergizing with IFN-γ is largely dependent on enhanced iNOS activity and NO production.

### TNF-α Synergizes with IFN-γ for Enhancing iNOS Expression and Cm-growth Inhibition in Cm-infected TC-1 Cells

In order to test whether IL-17A is the only cytokine which can synergize with IFN-γ for iNOS expression and anti-chlamydial activity, we examined the effect of TNF-α on IFN-γ mediated induction of iNOS expression and Cm-growth inhibition in TC-1 cells. As shown in Fig. 6, compared with IFN-γ stimulation alone, TNF-α (10 ng/ml) and IFN-γ (10 ng/ml) combination induced significantly higher iNOS mRNA expression (Fig. 6A and 6B) at 36 h after Cm infection, in parallel with increased inhibition of Cm growth (Fig. 6C). The results suggest that not only IL-17A but also TNF-α, and possibly some other cytokines may synergize with IFN-γ for enhancing iNOS expression and inhibiting Cm growth.

### IL-17A-neutralized Mice Exhibited a Significant Reduction on iNOS Expression

Our previous study has shown that IL-17A-neutralized mice led to more serious disease and in vivo chlamydial growth [14]. To test whether the neutralization of IL-17A has effect on iNOS expression in the lung, we further compared the expression of iNOS between IL-17A-neutralized mice and isotype control (IgG2a)-treated mice after Cm lung infection. As shown in Figure 7 A&B, IL-17A-neutralized mice exhibited significantly greater body weight loss and higher bacterial loads in the lung than IgG2a-treated mice at both day7 and day 13 following infection. Consistently, a significant reduction of iNOS expression in the IL-17A-neutralized mice was observed in comparison with IgG2a-treated mice at day 7 after infection (Fig. 7C and 7D), which further suggests that the inhibitory role of IL-17A on Cm growth involves enhancing iNOS expression.

## Discussion

iNOS-derived NO, as a potent antimicrobial agent, exerts profound cytotoxic effects on intracellular pathogens such as *Mycobacteria, Leishmania*, *Plasmodium*, *Toxoplasma* and *Chlamydia* [20], [21]. The present study has demonstrated a significant synergistic effect of IL-17A on IFNγ-mediated enhancement of iNOS expression and NO production, and inhibition of chlamydial growth in mouse lung epithelial and macrophage cells. We found that IL-17A significantly enhanced IFN-γ mediated increase of iNOS activity and NO production in mouse epithelial and macrophage cell lines and primary peritoneal macrophages in Cm infected cells, which was associated with enhanced inhibition of chlamydial growth. In addition, adding of AG (a potent inhibitor of iNOS) into the system significantly restored Cm growth. Further, neutralization of IL-17A in vivo reduced iNOS expression following Cm lung infection, which was associated with increased chlamydial growth in the lung.

It has been reported that protective *Chlamydia*-specific T-cell lines and clones can interact with infected epithelial cells to inhibit the growth of chlamydiae in an *in vitro* model, polarized epithelial-lymphocyte co-culture (PELC) system [1]. It was found in that study that the inhibition mainly correlated with high levels of IFN-γ secretion and NO production because neutralizing anti-IFN-γ antibodies and the NO synthase inhibitor largely reversed the inhibition [1]. Pulmonary epithelial cells are the principal target cells of chlamydial infection. After intranasal infection with Cm, cytokines such as IFN-γ, TNF-α and IL-17A can directly interact with pulmonary epithelial cells to inhibit intracellular Cm growth through inducing NO production. In the present study, we found that IL-17A have the ability to augment IFN-γ-mediated induction of iNOS expression in Cm-infected pulmonary epithelial cells and inhibition of Cm growth. Previous studies have shown that iNOS gene expression and subsequent NO production may be controlled by various agonists, such as TNF-α, IL-8, IL-1β and LPS, especially in combination with IFN-γ, in both mice and human [22]–[24]. Combination of IL-8 and IFNγ induces iNOS mRNA expression and functional enzyme production in cultured human keratinocytes [22]. Synergism of TNF-α and IFN-γ in inhibition of *C. pneumoniae* replication in vitro and in vivo was also reported [23]. Notably, LPS was also found to enhance iNOS expression in synergy with IFN-γ [24]. Since LPS is a common contaminant in recombinant proteins, the possible non-specific influence of LPS could be a concern in this type of study. However, the synergistic effect observed in the present study is unlikely due to contaminated LPS in the IL-17A preparation because the LPS level in the IL-17A is less than 0.1 ng per 1 µg of the cytokine, much lower than that used in the study showing the effect of LPS [24].

It was reported that IFN-γ mediated inhibition of *Chlamydia trachomatis* replication was mainly through inducing nitrite production in the murine macrophage cell line, RAW 264.7 [25]. In mouse peritoneal macrophages treated with IFN-γ, L-NMMA (an inhibitor of iNOS) inhibited nitrite production and partly restored Cm replication [25]. Murine macrophages have been reported to synthesize the obligatory cofactor tetrahydrobiopterin (BH4), essential for stabilization and function of the iNOS enzyme protein [26], although this has not been confirmed in human macrophages so far [27]. In the present study, we found IL-17A synergizes with IFN-γ in iNOS expression and NO production in macrophages. Consistently, we found that IL-17A synergizes with IFN-γ for inhibition of Cm growth in mouse peritoneal macrophages, which was significantly weakened by adding AG, an inhibitor of iNOS activity. Therefore, IL-17A synergizing with IFN-γ mediated NO production in macrophages is another important mechanism for inhibition of Cm replication.

Besides anti-microbial function, NO also acts as a regulator of the immune response, mainly by suppressing T cell activaty and function [9], [28]. Higher NO release by macrophage is known to induce immunosuppression and down-regulate Th1 cell development by inhibition of macrophage IL-12 synthesis and selective induction of apoptosis of activated Th1 cells [8], [19]. However, an overproduction of NO might cause abnormal inflammation and cellular damage [16], [28], which may augment the susceptibility of host against *Chlamydia* infection. It has been proposed that the quantity of NO released by infected macrophages is critical for balancing pathogenic and protective responses against chlamydial infection [19]. In the present study, we found that the inhibition rate of Cm growth was not increased by increasing the concentration of IL-17A when the cells were treated with 200 ng/ml IFN-γ (Fig. 5), suggesting that excessive production of IL-17A may not be helpful for controlling Cm infection. Therefore, an appropriate level of IL-17A production is important for host defense against chlamydial infection.

The demonstration of the synergy of IL-17A and IFN-γ in inhibiting chlamydial growth may have implication in rational vaccine development. Efforts have been made to enhance Th1 response in chlamydial infection by vaccination. The present data suggest that the combinational enhancement of both Th1 and Th17 responses may be a more efficient way to enhance host defense against chlamydial infections. However, it should be noted that some of the functions of IL-17A observed in mouse studies have yet to be confirmed in humans. Specifically, the role of IL-17A in human lung chlamydial infection remains unclear. Moreover, considering the potential toxic effect of NO, the significance of IL-17A-mediated NO production and its correlation with chlamydial inhibition should not be overemphasized. Therefore, it is important to further explore these issues in humans in the future. Notably, a T cell which produce both IL-17A and IFN-γ has been reported recently [29], [30]. Therefore, it seem reasonable to predict that this type of cells could be more efficient for the cooperation of IL-17A and IFN-γ than the different Th1 and Th17 cells for the inhibition of chlamydial growth. However, the possibility needs to be experimentally tested.

In conclusion, our study demonstrated that IL-17A can synergize with IFN-γ to inhibit intracellular Cm growth through enhancing intracellular iNOS expression. The finding provides new insight into the mechanism of the protective role played by IL-17A during intracellular pathogen infection, particularly *Chlamydia*. However, further studies are needed for using IL-17A as an immune adjuvant in chlamydial vaccination.
